# Multifactorial Imaging Analysis as a Platform for Studying Cellular Senescence Phenotypes

**DOI:** 10.3390/jimaging11100351

**Published:** 2025-10-08

**Authors:** Shatalova Rimma, Larin Ilya, Shevyrev Daniil

**Affiliations:** Translational Medicine Research Center, Sirius University of Science and Technology, Federal Territory Sirius, Olympic Ave. 1, 354340 Sirius, Russia

**Keywords:** cellular senescence, multimodal imaging, multiparametric analysis, stress-induced premature senescence, replicative senescence

## Abstract

Cellular senescence is a heterogeneous and dynamic state characterised by stable proliferation arrest, macromolecular damage and metabolic remodelling. Although markers such as SA-β-galactosidase staining, yH2AX foci and p53 activation are widely used as de facto standards, they are imperfect and differ in terms of sensitivity, specificity and dependence on context. We present a multifactorial imaging platform integrating scanning electron, flow cytometry and high-resolution confocal microscopy. This allows us to identify senescence phenotypes in three in vitro models: replicative ageing via serial passaging; dose-graded genotoxic stress under serum deprivation; and primary fibroblasts from young and elderly donors. We present a multimodal imaging framework to characterise senescence-associated phenotypes by integrating LysoTracker and MitoTracker microscopy and SA-β-gal/FACS, p16INK4a immunostaining provides independent confirmation of proliferative arrest. Combined nutrient deprivation and genotoxic challenge elicited the most pronounced and concordant organelle alterations relative to single stressors, aligning with age-donor differences. Our approach integrates structural and functional readouts across modalities, reducing the impact of phenotypic heterogeneity and providing reproducible multiparametric endpoints. Although the framework focuses on a robustly validated panel of phenotypes, it is extensible by nature and sensitive to distributional shifts. This allows both drug-specific redistribution of established markers and the emergence of atypical or transient phenotypes to be detected. This flexibility renders the platform suitable for comparative studies and the screening of senolytics and geroprotectors, as well as for refining the evolving landscape of senescence-associated states.

## 1. Introduction

Researchers initially considered cellular senescence to be a reaction to stress factors primarily associated with age-related changes and oncogenesis. It is now understood that cellular senescence is linked not only to development of tumours and the body’s natural immune response, but also to tissue regeneration, the regulation of fibrosis and the prevention of age-related diseases [1,2,3,4].

The senescent phenotype is marked by four hallmarks: cell-cycle arrest; macromolecular damage; metabolic dysregulation; and the acquisition of a pro-inflammatory, senescence-associated secretory phenotype (SASP) [5]. The variety of triggers reflects the wide range of stress signals that initiate senescence. For illustrative purposes, telomere erosion leads to replicative stress [6], while genotoxic double-stranded DNA damage activates the p53-p21 response [7]. Excessive accumulation of reactive oxygen species [8] and metabolic stress disrupt mitochondrial function and activate the p38 MAPK pathways [4,9,10]; RAS- or RAF-mediated activation protects tissues from tumours formation due to premature cell senescence [11,12]. There are other senescence triggers, including epigenetic changes in chromatin and endoplasmic reticulum damage and lysosome dysfunction. Each pathway yields a unique senescent phenotype, leading to substantial heterogeneity within and be-tween populations [13]. None of the variety of biomarkers covers the full range of phenotypes. There is a scientific consensus on a number of key features to be simulated using a combinatorial strategy [14,15]. Despite the widespread use of multi-marker molecular approaches for in-depth phenotyping of senescent cells, complex imaging platforms that can simultaneously capture the morphology and quantitative parameters of these cells are still needed.

There are three key reasons why it is important to reliably identify and quantify senescent cells. Firstly, it enables the early diagnosis and monitoring of age-related diseases. Secondly, it helps us to understand the regulatory role of senescent cells in normal physiological processes. Finally, it allows us to study the influence of various factors on cellular senescence processes by developing and verifying models to study geroprotectors, senolytics and senomorphs in vitro [16].

**Figure 1:**
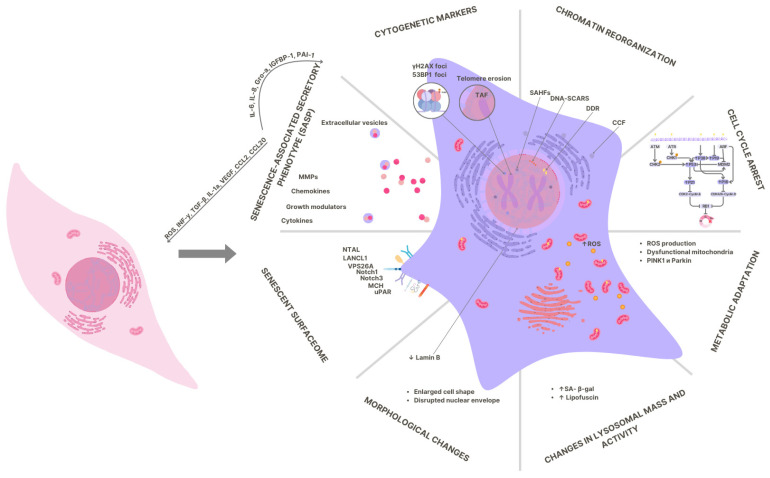
Key phenotypic markers of senescent cells (see diagram). Senescence forms as a set of interconnected, yet temporally distinct, modules: (1) Sustained DNA Damage Response (DDR). Early yH2AX/53BP1 foci and TIFs develop into long-lived ‘persistent’ DNA SCARS, indicating irreversible damage. Chromatin fragments (CCFs) appear in the cytoplasm, activating the cGAS-STING pathway and enhancing the SASP; (2) Reorganisation of chromatin and nuclear architecture. Chromatin reorganisation includes SAHF, as well as decreased lamin B1 and nuclear envelope defects; (3) Proliferative arrest. Supported by the p53-p21 and/or p16INK4a-RB axes, this is characterised by the rejection of G1/S and G2/M checkpoints; (4) Lysosomal-metabolic adaptation. The mass and activity of lysosomes increase (the source of the SA-β-Gal and GLB1 signals), mitochondrial function is impaired, and the level of ROS increases. Autophagy flux changes; (5) Morphology and adhesion. Typical features include an increase in cell size and flattening, an increase in actin stress filaments, and an enlargement of focal adhesions, which influence mechanosensitivity and migration; (6) Membrane profile of senescent cells. In some cell types, the expression of targetable membrane proteins increases, including DPP4/CD26 and uPAR (PLAUR). These markers are context-dependent and require validation for each model; (7) SASP and extracellular vesicles. Senescent cells secrete cytokines (e.g., IL-6/IL-8), growth factors and matrix metalloproteinases (MMPs).

The expected patterns from the literature must be emphasised. Senescence is accompanied by metabolic hyperactivation, with glycolysis and mitochondrial respiration increasing in parallel and β-oxidation of fatty acids and oxygen depletion rising. This reconfiguration meets the increased demand for ATP to support the SASP, chromatin remodelling and lysosomal autophagy (Figure 1) [17]. In senescent cells, pronounced lysosomal rearrangement is evident, including an increase in size and number of lysosomes, increased permeability of the lysosomal membrane (LMP), degree of acidification, lipofuscin accumulation, and lysosomal hydrolase hyperactivity [18,19,20]. The signal intensity of acidotropic fluorescent dyes has been shown to correlate with the volume and surface area of lysosomes. This allows them to be used as an indirect measure of organelle size in living cells [21,22].

Literature observations also suggest bimodal and multimodal distributions of lysosomal signals, which reflect the coexistence of resident lysosomes and enhanced autolysosomes [23]. As chronic stress and damage increase, such distributions shift and become more complex. This is consistent with a progressive increase in lysosomes and lysosomal enzymes, as well as de novo biogenesis, under conditions of senescence [24,25,26,27].

Mitochondrial dysfunction-associated senescence (MiDAS) is characterised by a decrease in respiratory capacity and membrane potential (Δψ_M_), accompanied by increased ROS production. MiDAS acts as both a trigger and a consequence of cellular senescence. This process is driven by the activation of the AMPK-p53 axis and the disruption of mitophagy via the PINK1/Parkin pathway, as well as by the modification of the secretory phenotype due to leakage of mitochondrial DNA into the cytoplasm, which activates the cGAS-STING pathway. These processes form positive feedback loops that maintain the senescent phenotype [28,29]. However, quantifying the morphology and functional activity of mitochondria is challenging due to their significant heterogeneity in length and branching degree [30]. The Δψ_M_ is a crucial bioenergetic parameter that reflects the state of oxidative phosphorylation and replication conjugation. It is a characteristic feature of mitochondrial dysfunction during senescence [31].

This study focuses on three key paradigms: replicative senescence involving serial passage, the dose-dependent induction of a genotoxic agent against a serum-starved background, and the comparison of cells from donors of different ages. The aim is to conduct in-depth phenotyping in order to develop and verify a multimodal imaging approach combining scanning electron microscopy (SEM), confocal microscopy of lysosomes and mitochondria, and flow cytometric analysis. Each modality contributes complementary information to the multimodal framework. Fluorescence microscopy using LysoTracker/MitoTracker provides spatially resolved, live-cell-compatible readouts of lysosomal and mitochondrial remodelling at the single-cell level. However, this technique is sensitive to photobleaching and phototoxicity, as well as acquisition settings, and therefore requires careful normalisation and controls. Flow cytometry (FACS) offers high-throughput, quantitative, population-level statistics (e.g., SA-β-gal/C12FDG intensity distributions) with robust calibration. However, it lacks spatial context and detail of cell morphology. Furthermore, spectral analysis methods cannot validate each other’s results. SEM provides ultrastructural fidelity and direct visual correlates of membrane texture, vesiculation and cytoskeletal organisation. However, it relies on fixed samples and, in this study, is used qualitatively to corroborate and refine hypotheses. Overall, the strengths and limitations of these methods complement each other: fluorescence microscopy and FACS provide the core quantitative data, while SEM offers high-resolution structural information to inform interpretation and future morphometric extensions.

## 2. Materials and Methods

### 2.1. Cell Culture

Primary dermal fibroblasts were obtained from skin biopsies taken from two age groups of C57BL/6 mice: 4-month-old and 19-month-old. The mice were anaesthetised with zolethyl xylazine intraperitoneally to collect the tissue samples. Skin fragments measuring 6 × 6 × 2 mm were excised and washed twice in phosphate-buffered saline (PBS) containing a 1× penicillin-streptomycin solution (Sigma-Aldrich, Burlington, MO, USA). The dermis was then separated from the epidermis and subcutaneous tissue via fine dissection. The fragments were then mechanically macerated with scissors, precipitated and dissociated in 0.2% collagenase IV 1 mg/mL (Gibco, Grand Island, NY, USA) in DMEM/F12 medium (PanEco, Moskow, Russia) at 37 °C for three hours, with gentle stirring every so often. After incubation, the enzyme was inactivated using 100% FBS (Capricorn Scientific, Ebsdorfergrund, Germany). The suspension was filtered through a 40-micron mesh sieve, spun down for five minutes at 300 g and seeded into a 6-well adhesive plate. The cells were grown in an Igla medium modified with Dulbecco’s medium and supplemented with 10% embryonic bovine serum, L-glutamine (PanEco, Moskow, Russia), and a penicillin-streptomycin solution. The cells were kept at 37 °C in a humidified incubator with 5% CO_2_ and subcultured at ~80–90% confluence. After this, the cells were subjected to sequential passage at a ratio of 1:2–1:3 approximately every 3–4 days. All experiments were performed on three populations of fibroblasts: cells from a young donor (YD; aged 4 months; passages ≤ P6), cells from an old donor (OD; aged 19 months; passages ≤ P6) and cells that had undergone 20–22 serial passages (RS).

### 2.2. Analysis of Beta-Galactosidase in Relation to the Senescence Process

SA-β-Gal (senescence-associated β-galactosidase) catalyses the hydrolytic cleavage of β-D-galactosides, producing galactose and corresponding indole derivatives in the process. X-Gal (5-bromo-4-chloro-3-indolyl-β-D-galactopyranoside, SibEnzyme, Moskow, Russia) is used as a substrate to quantify enzyme activity. X-Gal was prepared at a concentration of 20 mg/mL by dissolving the powder in dimethylformamide (DMF), after which it was stored in a dark container at −20 °C until the staining solution was prepared. The final working concentration of X-Gal in the staining solution was 1 mg/mL and was added just before the reaction began. Catalytic hydrolysis of X-Gal produces a bluish-turquoise precipitate of 5-bromo-4-chloro-3-hydroxyindole, which can be visualised using a light microscope. Although SA-β-Gal is present in the lysosomes of most cells (with an optimal pH of approximately 4.0), the reaction is carried out at pH 6.0 to selectively detect senescent cells, as this minimises background signals [15]. An increase in SA-β-Gal staining intensity correlates with lysosomal enzyme accumulation in senescent cells. However, the marker’s specificity may decrease in the presence of inflammatory or other stress responses [32,33].

Before staining, the cells were washed twice with PBS, fixed with a 0.2% glutaraldehyde solution for 10 min at room temperature, and then washed thoroughly with PBS again. The reaction was then carried out in a buffer solution containing 2 mM citrate-phosphate buffer (pH 6.0), 50 mM K_3_[Fe(CN)_6_], 50 mM K_4_[Fe(CN)_6_], 5 mM NaCl, 1 mM MgCl_2_ and 20 mg/mL X-Gal (in DMSO) (SibEnzyme, Moskow, Russia). The cells were then incubated in a thermostat at 37 °C for 20–24 h. Once the reaction had finished, the cells were washed with PBS again and visualised using a AxioScope 5 microscope (Carl Zeiss, Oberkochen, Germany) in a single shooting session to eliminate variability in lighting and equipment settings.

The percentage of SA-β-Gal^+^ cells (S%) was manually calculated in at least five visual fields of three independent wells for each condition. Relative survival (V%rel) was defined as the ratio of the number of cells in each field after treatment to the average number of cells in the corresponding control. To account for the cytotoxicity of the different inducers and accurately compare their effectiveness, the selectivity index (SI) was introduced and calculated using the formula SI = S%/(100 − V%rel). SI reflects the ratio of the yield of induced senescent cells to the fraction of dead cells. This enables agents to be ranked not only by their ability to cause senescence, but also by the selectivity of their action. To enable scaling beyond manual counting, can be used a semi-automated image-analysis workflow and, when required, a high-throughput C12FDG-based flow-cytometry readout of SA-β-Gal activity. Quality control included plate-level negative/positive references and blinded duplicate fields, with thresholds and counts logged per batch.

### 2.3. Scanning Electron Microscopy

SEM studies were performed using a Crossbeam 550 (Carl Zeiss, Oberkochen, Germany) at an accelerating voltage of 1 kV and a beam current of 300 pA. Two detectors were used: SE2 and InLens. These were used to display surface topography and visualise subcellular structures, including the contour of the nucleus.

To prepare the samples, primary mouse dermal fibroblasts were grown on cover slips. The cells were fixed with a 0.5% glutaraldehyde solution in PBS at room temperature for 10 min, after which they were washed twice with PBS. The same slides were then incubated with an SA-β-Gal staining reagent at 37 °C in a humidified incubator with 5% CO_2_ for 20–24 h. After imaging the stained cells with an Axio Vert. A1 microscope (Carl Zeiss, Oberkochen, Germany), the samples were dehydrated sequentially in ethanol solutions of increasing concentration (10%, 20%, 30%, 40%, 50%, 60%, 70%, 80%, 90% and 96%), for 10 min each. The samples were then incubated in a mixture of hexamethyldisilazane (HMDS) and ethanol (1:1) for 10 min, followed by three further incubations in 100% HMDS (twice for 10 min, then overnight), until complete evaporation. Prior to entering the chamber, the SEM was coated with a thin (10 nm) layer of Au/Pd (80:20%) using Quorum Q150T S/E/ES Plus (Quorum, London, UK).

### 2.4. Models of Senescence Induction Stress (SIPS)

The cells were seeded into 24- and 48-well plates in DMEM/F12 medium containing 10% FBS, 2 mM L-glutamine and 1% penicillin/streptomycin (all from PanEco, Moskow, Russia), and then incubated at 37 °C and 5% CO_2_ until 60–70% fusion was achieved. The following agents were used for the dose-dependent induction of senescence: Doxorubicin (Doxo): 250, 350 and 450 nM; Cisplatin (Cis): 5, 10 and 20 µM; etoposide (Eto): 5, 10 and 20 µM; and bleomycin (Bleo): 10, 14 and 25 µM. The agents were added to a medium containing 1% FBS and incubated for 24 h at 37 °C and 5% CO_2_. After this incubation period, the medium containing the inducers was removed and the cells were washed twice with PBS. The cells were then incubated in DMEM-F12 containing 1% FBS for six days, with the medium updated every three days. The following control conditions were used: (1) continuous cultivation in 10% FBS for seven days (Control 10%), (2) continuous cultivation in 1% FBS without inducers for seven days (Control 1%), and (3) a matched control for each inductor (24 h in 10% FBS, followed by six days in 1% FBS without genotoxin). This multilevel comparison enables the contributions of sporadic, metabolic, and genotoxic senescence to be separated, and allows the relative cell survival and selectivity index for each agent to be correctly assessed.

### 2.5. LysoTracker RedtoMitoTracker Orange

To visualise the cell nuclei, the cells were incubated with 2 µg/mL of the Hoechst 33,342 dye (Sigma-Aldrich, Burlington, MA, USA) for 15 min. Some of the cells were then stained with 50 nM acidotropic LysoTracker^®^ Red DND-99 (Invitrogen, Carlsbad, CA, USA) in a culture medium containing 1% FBS for 30 min at 37 °C in 5% CO_2_. The remaining cells were incubated in a culture medium containing 1% FBS and 0.4 µM MitoTracker Orange CMTM Ros (Invitrogen, Carlsbad, CA, USA). After staining, the cells were washed three times with PBS and visualised immediately using a Carl Zeiss LCSM 980 (Carl Zeiss, Oberkochen, Germany) laser confocal microscope in two modes: an 86×/0.8 Plan-Apochromat lens was used for high magnification and detailed study of subcellular structures, and a 20×/0.5 EC Plan-Neofluar lens was used for overviews and evaluation of large fields of view. For LysoTracker^®^ Red, the 405 nm and 561 nm laser lines were used with an output power of 0.2% and 0.15% of the nominal value, respectively. For MitoTracker^®^ Orange, the 405 nm and 561 nm laser lines were used with output powers of 0.2% and 0.18%, respectively. To ensure comparability of fluorescence intensities, the detector and exposure parameters were kept the same for all samples in a given series.

### 2.6. Immunofluorescence Staining Protocol for p16^INK4a^ Validation of Cellular Senescence

Immunofluorescence sample preparation: The cells were washed twice with PBS, then fixed with a 4% paraformaldehyde solution in PBS for 10 min at room temperature. The fixed cells were then washed three times with PBS. The samples were then soaked in a 0.2% Triton X-100 solution in PBS for 10 min, after which they were blocked for 60 min at room temperature in a PBS solution containing 5% foetal bovine serum (FBS). Primary antibodies against Anti-CDKN2A/p16^INK4a^ antibody produced in rabbit (Sigma-Aldrich, Burlington, Massachusetts, MO, USA, SAB5700620, 1:150) were diluted in PBS-T (PBS + 0.3% Tween-20 + 1% FBS) and applied overnight at 4 °C. After three washes with PBS-T, the samples were incubated with goat anti-rabbit IgG H&L (Abcam, Cambridge, UK, ab150081, 1:300) for one hour at room temperature in the dark. Fluorescent images were obtained using an EVOS inverted fluorescence microscope (Thermo Fisher Scientific, Waltham, MA, USA) equipped with a 20×/0.75 NA air objective lens. Exposure and lamp settings were kept constant throughout experimental comparisons. Secondary-only and isotype controls were included in each staining batch. This immunostaining was performed to validate the senescent status of cell populations following treatment with senescence-inducing agents.

### 2.7. Quantitative Analysis of Fluorescent Images and Statistical Data Processing

All quantitative biological indicators were first checked for normality using the Shapiro–Wilk test. Depending on the results, the data were described as either the mean ± standard error of the mean (SE) when the distribution was normal, or the median and interquartile range (IQR). The unpaired Welch *t*-test was used to compare groups for parametric data without assuming equality of variances. Multiple group comparisons were performed using ANOVA, followed by post hoc analysis using the Dunn test. Correction for multiple comparisons was performed in multiple pairwise tests using the Benjamini–Hochberg (FDR) method. All calculations and visualisations were performed using GraphPad Prism 10.1.2 (GraphPad Software, Boston, MA, USA) and Python 3.8, with the SciPy v1.7, Statsmodels v0.13 and Matplotlib v3.5 libraries.

Raw fluorescence intensities were divided by the two-dimensional projected area of each cell, as measured via bright-field segmentation, to correct for variability in cell spreading and isolate changes in lysosomal content per cytoplasmic territory. Analysing at least 100 cells in each of the inducer and donor conditions meets the recommended threshold for single-cell fluorescence studies. All fluorescence images were acquired using an LSCM 980 confocal microscope (Carl Zeiss, Oberkochen, Germany) and saved as single-plane extended depth of focus (EDF) CZI files. The array was extracted using the czifile Python library. For four-dimensional datasets, the first channel was summed over z; for three-dimensional datasets, the first channel was used directly [34].

Binary cell masks were drawn manually and exported as PNG files. The mask was resized to match the dimensions of the fluorescence array using nearest-neighbour interpolation and converted to a Boolean array, where white pixels denote the cell contour. Physical pixel dimensions (µm) were read from the CZI metadata. When both X and Y distances were available, the mean of the two was taken as the isotropic pixel size.

The total number of pixels in the mask was multiplied by the squared pixel size to yield the projected cell area in µm^2^. All raw fluorescence values within the mask were then divided by this area to give an intensity density (arbitrary units per µm^2^).

Normalised intensities were binned into 30,000 log-spaced intervals between zero and the maximum observed value. Counts per bin were then averaged across 95–600 cells (depending on the experiment) to yield counts per bin per cell. The resulting distribution was plotted on log-log axes (intensity density versus average counts) using Matplotlib. Identical axis limits and plotting parameters were used for all conditions to allow direct comparison. All these steps were implemented in a reproducible Python pipeline using the czifile, PIL and NumPy libraries to ensure consistent and unbiased estimation of projected cell area across all experimental conditions.

Analysis of the inflexion points of LysoTracker DND-99 fluorescence intensity distribution histograms reveals several areas of varying curvature, indicating multimodal data distribution. Such multimodal profiles may reflect lysosome population heterogeneity in terms of size or composition [35]. However, the direct correlation between inflexion points and organelle sizes requires confirmation through direct morphological measurements, such as SEM analysis or immunofluorescence microscopy, in order to separate subpopulations. In the case of LysoTracker, this could indicate different lysosome densities or sizes, but the analysis always considers background signals, photobleaching and detection thresholds.

To emphasise the differences in the shoulders and tails of the distributions, the axes were log-transformed. The influence of donor age, inductors, and peak order on local maximum position was estimated (Equation (1)) using a linear mixed model (LMM):

(1) $${log}_{10}\left(X_{peak}\right)\;\sim \;C_{donor\;}+\;C_{inducer}+\;{peak}_{id}+\;\left(1|curve\right),$$

where C_donor_ and C_inducer_—categorical factors representing the donors (YD, OD and RS) and inductors (Control FBS 1% (C1), Doxo, Cis, Eto and Bleo), as well as the ordinal number of the peak (1, 2, etc.). (1∣curve)—a random intercept at the curve level, accounting for the intra-curve dependence of several peaks.

The inductor coefficients were interpreted as an offset relative to C10. Maximum likelihood was based on two-way *p*-values, standard errors and 95% confidence intervals for fixed effects. The normality of the residues was tested using the Shapiro–Wilk test. Homogeneity of variances was tested using the Levene test for C_donor_ group.

A multi-sample Anderson-Darling test was used to compare the distribution shape of peak positions between donors {log10(X_peak_)} within each inductor. The test only included inductions where there were ≥2 peaks in each subgroup. The test yielded statistics and an approximate *p*-value. Since there may be several peaks in one curve, the AD test uses potentially dependent observations within the curve. This risk of pseudo-replication is offset by the fact that main factor inference is based on the LMM, which includes a random intercept (1∣curve).

In order to decouple the Δψ_M_ from differences in cell size, and thus compare mitochondrial function rather than volume, the integrated fluorescence of each cell was divided by its measured area. This yielded a mean fluorescence per pixel for each cell, reflecting Δψ_M_ density independently of cell morphology.

We summarised the MitoTracker Orange intensity per cell area (X, a.u./µm^2^) as a cumulative distribution function (CDF; 0–100% of cells). For log-axis analysis, the CDFs were expressed as α(u), where u = log_10_(X_peak_). Data-driven inflexion points (θ_M_) were defined as the local maxima of dα/du, partitioning each CDF into consecutive segments: [min, θ_1_], [θ_1_, θ_2_], and [θ_2_, max]. The CDF was divided into consecutive zones based on the maximum points. The conversion contribution (Δα) and the *X*-axis width were then calculated for each zone: Δψ_1_ = α(θ_1_), Δψ_2_ = α(θ_2_) − α(θ_1_), and Δψ_3_ = 100 − α(θ_2_). These three percentages sum to 100% for every curve.

The full set of normalised intensities was partitioned into four bins using these thresholds: depolarised (Δψ_1_), mid (Δψ_2_), and hyperpolarised (Δψ_3_). The proportion of cells in each bin was calculated as a percentage of the total. These four percentages can describe shifts between depolarised, intermediate, and hyperpolarised subpopulations in response to nutrient or genotoxic stress, thereby illustrating the mitochondrial heterogeneity of the population under each experimental condition.

## 3. Results and Discussion

### 3.1. A Quantitative Assessment of SA-β-Gal Activity in Cells of Different Ages and During Replicative Depletion

We employed several induction paradigms, including replicative senescence through passaging (RS), and a comparison of fibroblasts from young (YD) and old (OD) donors subjected to dose-dependent genotoxic effects against a serum-starved background. This approach allowed us to identify common signs of ageing independent of the trigger type, pinpoint specific phenotypes for individual stress response pathways and accurately rank inducers by effectiveness while considering cytotoxicity using the selectivity index, in accordance with descriptive statistical theory.

The multimodal design of the experiment is important for two reasons. On the one hand, it reduces the risk of misinterpretation associated with single markers by ensuring consistent independent measurements (from organelles to populations). On the other hand, it reveals the context-dependent architecture of the phenotype: the combination of serum deficiency and genotoxins results in the most severe and complex organelle disorders, whereas replicative senescence and donor age result in milder yet stable basal shifts.

**Figure 2:**
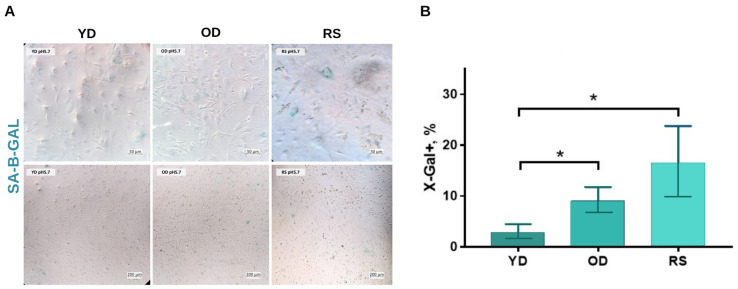
(**A**) Representative bright-field images showing SA-β-gal staining in the following in vitro models: early passage from a young donor (YD); early passage from an old donor (OD); and replicatively senescent cells (RS). Scale ruler: 50 µm (**lower**), 200 µm (**upper**). (**B**) Quantification of SA-β-gal staining. One-factor multifactorial analysis of variance was used for statistical analysis. * *p* < 0.05.

SA-β-Gal crosslinking was performed on three populations of fibroblasts: cells from a YD, OD and RS. Total β-Gal activity at pH 4.3 was determined as a positive control (Figure 2).

The proportion of SA-β-Gal-positive YD cells was 2.5% ± 0.8%, which is characteristic of highly proliferating fibroblasts [36]. In the OD population, the percentage of stained cells increased to 9.1% ± 1.2% (*p* < 0.05 vs. YD). The cells exhibited thickening of the cytoplasm and an increased nuclear volume. In the RS culture, SA-β-Gal positivity reached 16.3% ± 2.5% (*p* < 0.05 vs. YD and OD). Along with increased size, the fibroblasts demonstrated pronounced vacuolisation and the formation of cytoplasmic aggregates. An intergroup comparison using the single-factor ANOVA method revealed statistically significant differences in SA-β-Gal activity levels.

These data confirm that both age-related and replicative stress lead to an increase in cellular senescence due to the accumulation of intracellular stress and metabolic disorders [37]. Valieva et al. demonstrated that fibroblasts from elderly donors have a significantly higher proportion of SA-β-Gal-positive cells than those from young donors, all other things being equal [38]. Martinez-Zamudio et al. showed a similar relationship in immune T cells using quantitative fluorescence detection of β-galactosidase [39]. Although SA-β-Gal remains a recognised marker of cellular senescence, the classical histochemical technique predominantly provides high-quality results. However, the lack of a clear staining threshold makes it difficult to accurately identify cells with moderate beta-galactosidase activity [32,40].

### 3.2. The Differential Effects of Ageing Inducers on the Activity of the Beta-Galactosidase Enzyme

**Figure 3:**
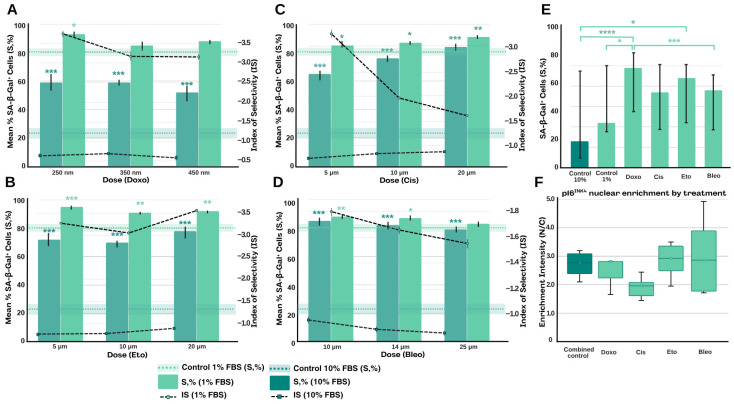
(**A**–**D**) Comparison of the induction of cellular senescence (SA-β-Gal^+^%) and selectivity (IS) in the presence of Doxorubicin (Doxo) (**A**), Cisplatin (Cis) (**B**), Etoposide (Eto) (**C**) and bleomycin (Bleo) (**D**) at 10% and 1% serum in the medium. The columns represent the mean proportion of SA-β-Gal^+^ cells ± SE, and the horizontal lines and shaded areas correspond to the control mean proportion ± SE. Statistically significant differences from the control are marked with asterisks (* *p* < 0.05, ** *p* < 0.01, *** *p* < 0.001; Welch’s *t*-test). (**E**) Flow cytometry (MFI ± IQR) for quantifying SA-β-Gal activity in control and induced populations. (Dunn’s comparison test: * *p* < 0.05, *** *p* < 0.01, **** *p* < 0.0001; each group >10). (**F**) p16^INK4a^ nuclear enrichment by treatment is expressed as the nuclear-to-cytoplasmic fluorescence ratio (enrichment = mean nucleus intensity/mean cytoplasm intensity). Boxplots show the per-image distribution (median and IQR) with the individual image points overlaid; the points indicate the group medians.

We analysed four classical inducers of senescence (Doxorubicin, Cisplatin, Etoposide and Bleomycin) in an in vitro dose-dependent model of genotoxic stress induction under serum starvation conditions at two concentrations (10% and 1%). Under 10% serum conditions, all four agents caused a statistically significant increase in the proportion of SA-β-Gal^+^ cells compared to the control group (Welch’s *t*-test, *p* < 0.05). The highest S% values (up to ~80–85%) were achieved with average doses of cisplatin (Figure 3C) and bleomycin (Figure 3D) (10 µM and 14 µM, respectively). In contrast, doxorubicin (Figure 3A) and etoposide (Figure 3B) only caused a moderate increase in S% (up to ~55–65%), alongside a high survival rate (V%rel > 85%). At 10% FBS, cell viability did not significantly impact the induction of senescence, resulting in a low selectivity index for all agents.

Decreasing the serum concentration to 1% resulted in an increase in the baseline S% level in the control group to 80–90%, limiting the potential for further increases in S%. Comparing SA-β-Gal activity (Figure 3E) and selectivity at serum concentrations of 10% and 1% allows the combined effect of starvation and genotoxic agents to be assessed. Decreasing the serum concentration from 10% to 1% increases the selectivity index (SI) of all four inducers from below one to a range of 1.66–3.69. This indicates an increased induction of cellular senescence under metabolic stress conditions.

Quantitative analysis of nuclear-to-cytoplasmic (N/C) enrichment intensity of p16^INK4a^ across different treatments (Figure 3F). Combined control cells displayed stable enrichment levels around 3.0. Doxorubicin (Doxo) and cisplatin (Cis) reduced nuclear accumulation, with Cis showing the strongest decline to 1.8 N/C. In contrast, etoposide (Eto) maintained enrichment comparable to controls, while bleomycin (Bleo) induced a marked increase in variability, with a subset of cells showing the highest enrichment intensities (>4.0). These patterns indicate treatment-specific modulation of p16^INK4a^ nuclear partitioning, with bleomycin uniquely enhancing heterogeneity of response.

A comparative analysis of serum starvation with genotoxins revealed that doxorubicin and etoposide exhibited the best selectivity (high yield of senescent cells with moderate loss of viability), whereas cisplatin and bleomycin exhibited a weaker induction-to-cytotoxicity ratio.

We demonstrated that combining serum starvation with genotoxic stress results in the induction of cellular senescence in a dose-dependent manner, with selectivity profiles that depend on the nature of the inducing agent. Serum starvation activates the AMPK-mTOR signalling axis and initiates autophagy, creating a metabolic environment in which DNA repair and cell cycle restoration are limited [41]. Metabolic stress increases ROS production and depletes antioxidant systems, creating conditions in which DNA breaks accumulate during treatment with genotoxins such as doxorubicin, etoposide, cisplatin and bleomycin [42]. Doxorubicin and etoposide demonstrate the greatest increase in IS due to their ability to provoke not only double DNA breaks, but also enhance ROS stress during starvation. Cisplatin and bleomycin also benefit from metabolic stress, albeit to a lesser extent, by inducing cross-links and Fenton-mediated breaks. Thus, starvation creates an environment in which cells are more sensitive to DNA damage, and the selectivity of different inducers (intercalation/topoisomerase vs. crosslinks/radicals) depends on their mechanisms. Our data are consistent with published reports on specific sensitivities to different types of DNA damage [43,44] and highlight that cisplatin and bleomycin reliably induce persistent senescence. In contrast, doxorubicin and etoposide are more effective at studying early and partial senescence phenotypes. Multi-marker molecular analysis and dynamic monitoring at different time points would provide further clarification of the peripheral mechanisms of reversibility and fixation of the senescence phenotype.

SA-β-Gal has long been recognised as a fundamental marker of senescent cells [15]. However, the intensity of the turquoise precipitation can be affected by the conditions of the incubation, the concentration of the solution and the pH, potentially leading to false positive or negative results in cultures undergoing quasi-ageing or exhibiting high fluorescence [32]. The lack of a standardised criterion for the ‘threshold’ intensity of staining reduces the objectivity of counting cells with low or moderate SA-β-Gal expression, particularly when populations with a similar level of senescence are being compared. To obtain more objective quantitative indicators and minimise subjectivity when selecting the threshold for cells with low and moderate β-galactosidase activity, it is advisable to combine SA-β-Gal staining with fluorescent flow cytometry (FACS) analysis and digital image processing. This increases the reproducibility and accuracy with which populations of senescent cells can be compared.

Figure 3B shows the results of a quantitative flow cytometry analysis using the C12FDG kit. Monitoring at 10% FBS (C10) shows a low baseline signal level. Moving from C10 to C1 doubled the MFI (×1.5–2), indicating an increase in the basal β-galactosidase signal under starvation conditions. Doxorubicin and etoposide produced a moderate increase in MFI (approximately 2–3-fold), whereas cisplatin and bleomycin produced a more pronounced increase (approximately 3–4-fold) compared to C10. This is consistent with the cytochemistry data, which show that cisplatin and bleomycin produce a higher percentage of ‘senescent’ cells. The wide range of MFI values (high IQR) is particularly evident in the cisplatin and bleomycin groups, reflecting heterogeneity in the population of SA-β-Gal-positive cells. In contrast, the distribution is narrower for doxorubicin and etoposide, indicating a more uniform, albeit less intense, response.

### 3.3. Identification and Morphology of Senescent Cells

Cellular senescence is accompanied by profound remodelling of the cytoskeleton and adhesive properties, which alters the cell’s mechanosensitivity to matrix signals. Typical changes include an increase in cell area and flattening, as well as vacuolisation, polynucleation and enlargement of focal adhesions. These changes enhance adhesion between the cell and the underlying surface, thereby slowing migration [45,46].

**Figure 4:**
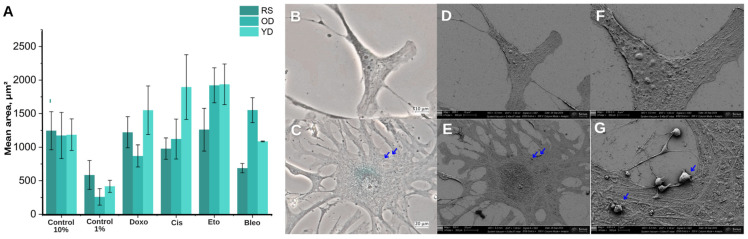
Correlative morphology of fibroblasts. (**A**) Distribution of cell surface areas in control and SISP-treated fibroblasts. The cell areas of three in vitro models were quantified from confocal images 7 days post-treatment with Doxorubicin (250 nM), Cisplatin (5µM), etoposide (5µM) or bleomycin (10µM). The histograms show the percentage of cells (n = 150–500 per condition), illustrating a pronounced shift towards larger cell sizes upon induction of senescence. (**B**,**C**) Bright-field X-Gal images: a non-senescent cell is shown at 20× magnification in (**B**); an SA-β-Gal^+^ cell is shown at 20× magnification in (**C**). (**D**,**E**) SE images of the same cells as in (**B**,**C**). (**F**,**G**) High-magnification SE (4000×) of the cell surface: blue arrows indicate cytoplasmic vesicles. Scale bars: 20 µm (**C**,**E**), 10 µm (**B**,**D**), 4 µm (**F**), 2 µm (**G**).

Pronounced abnormalities in the morphology of SA-β-Gal-positive cells were observed. Their area increased significantly compared to the untreated control (Figure 3A). Careful examination of the intercellular contacts of SA-β-Gal-positive cells revealed uneven contours and strongly outlined membrane protrusions, as well as stretched filopodia forming a lace-like structure (Figure 4C). These changes indicate a difference in adhesive properties compared to SA-β-Gal-negative fibroblasts (Figure 4B,D,F).

The images (Figure 4E) show the flattened, outstretched shape of a senescent cell. These cells are characterised by an enlarged projection area and an irregular membrane contour. This corresponds to the weakening of cell-to-cell and cell-to-matrix contacts that occurs during senescence. The cell surface exhibits pronounced roughness with numerous bumps and microvesicles, which are classic features of senescent cells visible throughout the field of vision (Figure 4G).

In the region of the perinuclear space, electron-dense material accumulates to form an elevated, localised belt around the nucleus (Figure 4E). This is most likely due to endoplasmic reticulum overload in this region and the expansion of the perinuclear lysosomal pool, which are typical signs of ER stress and increased autophagolysosomal metabolism during senescence. Thin lamellipodia and enlarged filopodia bearing single vesicles are visible along the cell’s periphery. The true senescence phenotype is confirmed by the abundance of vesicles and the expanded membrane bulges, which are seen together in these topographic features.

### 3.4. Area-Normalised Cells Fluorescence Histogram Profiling with Inflexion-Point Detection

Intracellular lysosome populations are inherently heterogeneous. When stained with LysoTracker, the number, position and amplitude of peaks in each cell’s histogram can be different. These peaks correspond to different lysosome subpopulations, such as small homeostatic organelles and larger compartments. This diversity may result from senescence or be induced by stress. A standard fixed-effects model would either require us to discard curves with fewer peaks, or ignore dependency between peaks within the same curve. Fortunately, the LMM treats each detected peak as an observation and explicitly accounts for the correlation between peaks from the same curve (random intercept). It also simultaneously estimates the proportion of variation in peak position driven by donor age, inducer type and the tendency for successive peaks to occur at higher intensities using the peak as the covariate. In biological terms, the random intercept captures variability between curves (e.g., slight differences in cell confluence, staining efficiency, or imaging settings), while the fixed effects quantify systematic shifts.

**Figure 5:**
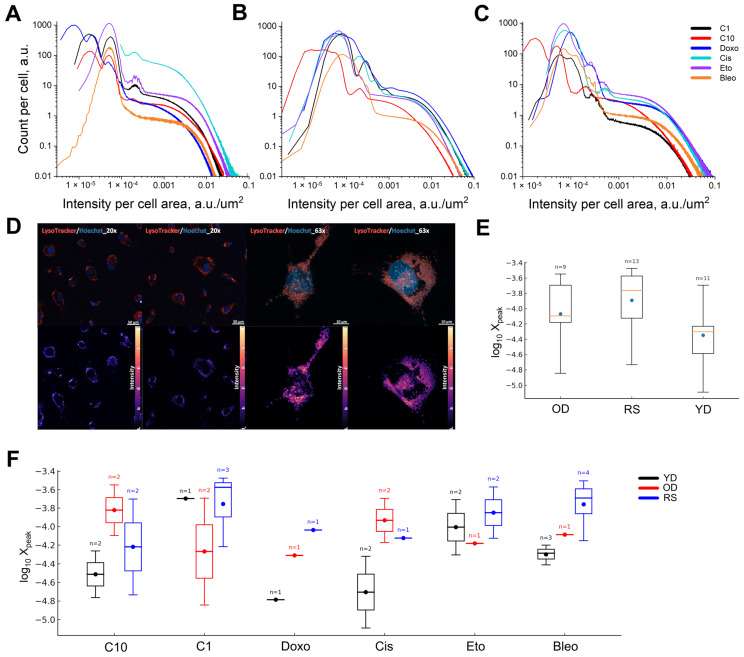
Normalised LysoTracker distributions by cell area reveal donor- and stress-dependent rearrangement of lysosomal subpopulations. (**A**–**C**) Histograms showing the number of events per cell versus the intensity per unit area of the cell on a log-log scale: (**A**): young donor (YD); (**B**): old donor (OD); (**C**): replicatively senescent cells (RS). At least 100 cells were used for each condition. (**D**) Representative confocal micrographs of LysoTracker/Hoechst control-doxorubicin pairs (20× and 63×). The intensity scales are pseudocolours (bottom scale from 0 to 255 normalised). (**E**) A summary box diagram showing the positions of local maxima by donors (OD, RS and YD; the points represent the averages and n represents the number of peaks). Local maxima were distinguished on the raw curves by prominence and minimum distance. The *x*-axis is the coordinate of the peak on a log scale. (**F**) Group box diagrams for inductor-donor pairs in one row. The points are the averages and n is the number of peaks in the group.

Log-log analysis of the transformed histograms of LysoTracker for groups (YD, OD and RS) and conditions (Cotrol FBS 1% (C1), Cotrol FBS 10% (C10), doxorubicin, cisplatin, etoposide and bleomycin) revealed 34 local maxima (Figure 5A–C). Compared to YD, the peak positions in OD/RS shift towards higher log_10_X_peak_ values. This is consistent with the accumulation of enlarged lysosomal compartments during ageing and replicative senescence. Compared to C10, conditions produce additional shifts, which are most noticeable under starvation conditions (C1) and in the presence of DNA-damaging agents. Furthermore, we emphasise that each subsequent local maximum occurs at higher log_10_X_peak_ values, reflecting the transition from small/medium to large lysosomes.

One to three peaks were found in each donor-inducer combination, indicating the presence of several lysosomal subpopulations of different volumes and/or acidities. To quantify the shifts in peak positions a LMM was constructed using the maximum likelihood method. The shift in the medians OD and RS clearly reflects the appearance of larger lysosomes (Figure 5E). Genotoxic drugs cause the greatest peak shifts towards high intensities (large lysosomes), particularly in OD and RS cells (Figure 5F). The data do not deviate significantly from normality (Shapiro–Wilk: W = 0.9553, *p* = 0.1899), and group variances are homogeneous across donors (Levene’s test: F = 0.1757, *p* = 0.8397).

This confirms the validity of the parametric conclusions. To evaluate variations in the shape of the distribution between donors at each exposure level, we used the Anderson-Darling multi-sample test (AD). This test detects shifts in the centre, as well as changes in the shoulders and tails of the distribution. Bleomycin showed the greatest discrepancy between young (YD), old (OD) and replicatively depleted (RS) cells (A^2^ = 3.12, *p* = 0.017). This suggests restructuring of the entire peak curve, with an increase in the right tail and emergence of additional local maxima in the OD/RS groups. This is consistent with heterogeneous lysosome enlargement and increased acid load during oxidative damage. Cisplatin showed a tendency to differ between the YD and OD groups (A^2^ = 1.54, *p* = 0.075), which is consistent with a partial redistribution of subpopulations (possibly due to drug sequestration and mitophagy, leading to an increase in the lysosomal lobe). Under conditions C1 and C10, the distribution shapes did not differ statistically between donors (*p* = 0.25 in both cases), indicating that basic nutrition alone does not alter the architecture of lysosomal subpopulations after normalisation to the cell area. The previously observed differences in medians primarily reflect a shift in peak position (as confirmed by LMM), rather than shape. No shape differences were found for etoposide either (*p* = 0.25).

Note that the AD test was only performed where there were ≥2 peaks in each donor group. Therefore, doxorubicin was excluded from this analysis due to a our approach operationalises n-insufficient number of peaks in one of the groups. In our operationalised approach, we did not implement a dedicated novelty/out-of-distribution (OOD) detector (e.g., conformal prediction, one-class models). Instead, low-confidence cases are treated conservatively as unassigned and routed to confirmatory assays (e.g., alternative markers or replicate measurements) rather than being force-classified. This study focuses on characterisation of predefined senescence-associated phenotypes. This reduces the risk of over-interpretation while ensuring that the panel can be expanded in future. It should be noted that the given *p*-values are not adjusted for multiple comparisons. With FDR control at 0.10, the result for bleomycin remains significant; however, with FDR control at 0.05, the result is only marginal. When used alongside the LMM, which records shifts in peak location, the results of the AD test show that bleomycin induces qualitative changes in distribution shape, whereas quantitative shifts in peak position dominate other effects without pronounced changes in subpopulation architecture. Meanwhile, the differences between YD, OD and RS manifest as shifts in the centre, while individual inductors (e.g., Bleo) cause a rearrangement of the shape without shifting the centre.

LysoTracker multimodal histograms likely indicate the presence of both resident lysosomes and stress-induced autolysosomes. The left- and right-sided weighting tracks changes in autophagy and lysosomal biogenesis, respectively. Δψ_M_ segment CDF analysis enables the population to be divided into depolarised, medium-polarised and hyperpolarised groups, indicating the state of mitochondrial quality control (recognition of mitochondrial damage, Parkin-mediated labelling and compensatory hyperpolarisation). Therefore, we interpret OD/RS shifts to the right, as well as doxorubicin-induced shifts to the left, as evidence of coordinated remodelling of lysosomes and mitochondria in response to genotoxic/metabolic stress. However, we emphasise that definitive causal relationships require joint ultrastructural or immunomarker verification.

**Figure 6:**
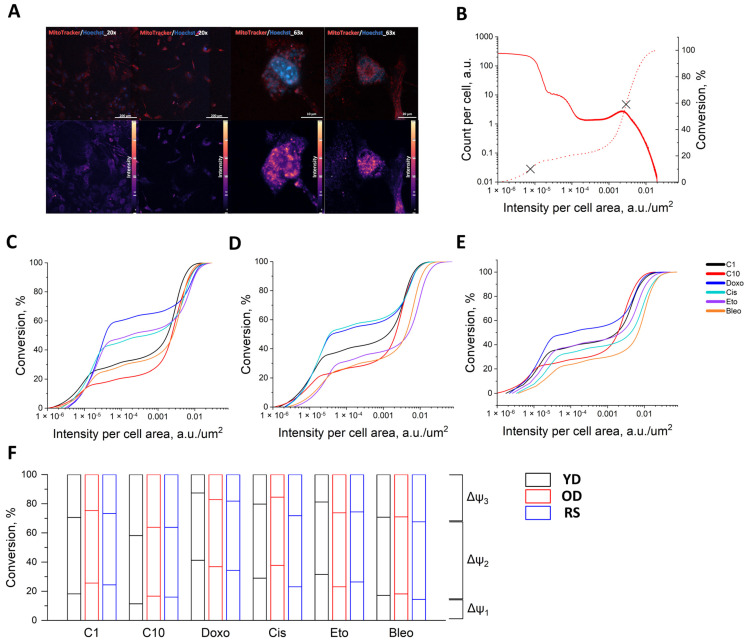
(**A**) Representative confocal micrographs of MitoTracker/Hoechst control-doxorubicin pairs (20× and 63×). The intensity scales are pseudocolours (bottom scale from 0 to 255 normalised). MitoTracker intensity distribution analysis: (**B**) The initial log-log curve for YD-C10 and the corresponding cumulative distribution function (count per cell: solid line; conversion curve: dashed line) (**C**,**D**,**F**) curve by donors. The characteristic points (θ_M_) are marked with crosses. (**C**–**E**) The CDF intensity for all inductors (YD, OD and RS from left to right).

Bimodal MitoTracker intensity histograms were observed in young (YD), old (OD) and replicatively senescent (RS) fibroblasts (Figure 6B). The conversion distribution function (CDF) curves were sigmoidal across all donor-inducer combinations (Figure 6C–E). To enable direct, model-free comparisons, we quantified conversion fractions in three segments defined by the data-driven inflexion points: the low-intensity segment [0, θ_1_] (Δψ_1_), mid-intensity segment [θ_1_, θ_2_] (Δψ_2_), and the high-intensity segment [θ_2_, 100] (Δψ_3_). Group differences are thus interpreted purely as redistribution of percent conversion among these segments (stacked bar plots in Figure 6F): increases in Δα_3_ reflect a larger fraction of cells occupying the high-potential range, whereas increases in Δα_1_ indicate expansion of the low-potential pool. This percent-based summary is invariant to vertical scaling of the histograms and directly comparable across donors and inducers.

A comparison of conversion curves (CDFs) revealed that genotoxic inducers redistribute the proportion of the population between low-, intermediate- and high-intensity zones to varying degrees. Compared to several conditions (C10, Doxo and Cis) consistently shifted the distribution to the left in all donors, increasing Δψ_1_ while compensatorily decreasing Δψ_3_. This effect was most pronounced in YD (Doxo: +30, +28; Cis: +17, −21), comparable in OD (+20, −18; +21, −20), and slightly less so in RS (+18, −18; +7, −7). The fraction in Δψ_2_ varies minimally during these treatments, usually by about four percentage points, indicating predominant mass transfer between opposite states. Etoposid also increases the low-intensity fraction, albeit more moderately: +20 pp for Δψ_1_ and −22 pp for Δψ_3_ in YD and +10 pp for Δψ_1_ and −10 pp for Δψ_3_ in RS. Δψ_2_ remains close to C10. C1 with respect to C10 causes a slight left shift and expansion of the mid-intensity zone: +6 pp for Δψ_1_ and −12 pp for Δψ_3_ in YD, −11 pp for Δψ_1_ in OD and +8 pp for Δψ_2_ and −9 pp for Δψ_1_ in RS. Collectively, these results show that Doxo/Cis mainly increases the proportion of low-potential cells due to the hyperpolarised tail; Bleo mainly increases the proportion of cells in the intermediate-intensity zone; and C1 causes mild but directed changes. All conclusions are expressed as percentages and are therefore directly comparable between donors and inducers. We consider a comparison with differences of less than or equal to 5% to be statistically insignificant.

These data reveal that replicative senescence (RS) results in the greatest heterogeneity in Δψ_M_ in response to both nutrient and genotoxic stress. This manifests as clusters that are simultaneously depolarised and hyperpolarised. In contrast, cells from an old donor exhibit low heterogeneity in mitochondrial junctions, resulting from the same stressors. Cells from young donors (YD) demonstrate a strong capacity for hyperpolarisation, but contain fewer low-Δψ_M_ subsets. This layered divergence highlights the interaction between donor age and passaging in shaping the landscape of the mitochondrial membrane potential under stress.

Mechanistically interpreting the effects and reproducing them on different donors separates the phenotype from inductor-specific signatures and creates a reliable, multiparametric platform for screening senolytics and geroprotectors. Notably, the combined low-serum and genotoxic effects resulted in the most significant alterations to the lysosomal-mitochondrial architecture. Meanwhile, the replicative and age-associated models caused more moderate, yet coherent, biases in the same parameters. These findings highlight the importance of selecting an induction model and employing distributional analsis to accurately interpret multi-marker phenotypes of senescence.

## 4. Conclusions

In this study, we present an integrated imaging platform combining ultrastructural, confocal and flow cytometry techniques to characterise cellular ageing with unparalleled precision at the organelle level. By focusing on the morphofunctional characteristics of mitochondria and lysosomes, such as ΔθM heterogeneity and lysosomal remodelling, we reveal the multidimensional heterogeneity of senescent cells. This methodological framework provides a reproducible, quantitative basis for evalu-ating the effectiveness of new senotherapeutic agents, such as senolytics and senomorphics. By using multiparametric phenotyping, it avoids the limitations of single-marker approaches.

We also recognise the strong potential of combining imaging-derived metrics with emerging computational tools, such as DeepScence, a deep-learning method designed to identify senescent cells in single-cell and spatial transcriptomic datasets [47]. Although it is currently only available as a preprint, it demonstrates how integrating visual and omics data can improve our understanding of cellular ageing.

The operational scope of this framework is the characterisation of a set of senescence-associated phenotypes. While fluorescence microscopy and flow cytometry form the quantitative core, SEM provides qualitative ultrastructural corroboration rather than primary quantitative data. We do not profess to offer comprehensive coverage or automated identification of novel or transient phenotypes. These tasks are reserved for orthogonal validation through visualisation and future methodological extensions, including systematic SEM morphometry and cross-site harmonisation. In the future, combining our imaging techniques with AI analysis and multiomics, including deep learning, will allow us to generate precise, quantitative in situ models of the ageing phenotype. This approach is in line with the increasing focus on reproduci-bility and interdisciplinary integration within the field of cellular ageing.

## Data Availability

The data presented in this study are available on request from the corresponding author due to privacy.
